# Medical history of coronary artery disease and time to electrocardiogram in the emergency department: a real-life, single-center, retrospective analysis

**DOI:** 10.1186/s12872-021-02274-1

**Published:** 2021-10-07

**Authors:** Lukas Andreas Heger, Tina Glück, Klaus Kaier, Marcus Hortmann, Marina Rieder, Patrick M. Siegel, Philipp Diehl, Tobias Wengenmayer, Christoph B. Olivier, Christoph Bode, Hans-Joerg Busch, Daniel Duerschmied, Ingo Ahrens

**Affiliations:** 1 Department of Cardiology and Angiology I, Heart Center Freiburg University, Faculty of Medicine, University of Freiburg, Freiburg, Germany; 2 Institute of Medical Biometry and Statistics, Faculty of Medicine and Medical Center, University of Freiburg, Freiburg, Germany; 3 Center of Big Data Analysis in Cardiology (CeBAC), Heart Center Freiburg University, Department of Cardiology and Angiology I, Faculty of Medicine, University of Freiburg, Freiburg, Germany; 4 Departement of Emergency Medicine, University Medical Center Freiburg, Medical Faculty, University of Freiburg, Freiburg, Germany; 5 Department of Cardiology and Medical Intensive Care, Augustinerinnen Hospital, Academic Teaching Hospital University of Cologne, Cologne, Germany

**Keywords:** Cardiac Evaluation, Coronary artery disease, Chest pain unit, Door-to-ECG time, Door-to-coronary-angiography, Emergency department

## Abstract

**Background:**

Timely acquisition of 12-lead Electrocardiogram (ECG) in the emergency department (ED) is crucial and recommended by current guidelines.

**Objectives:**

To evaluate the association of medical history of coronary artery disease (hCAD) on door-to-ECG time in the ED.

**Methods:**

In this single center, retrospective cohort study, patients admitted to ED for cardiac evaluation were grouped according to hCAD and no hCAD. The primary outcome was door-to-ECG time. A multivariate analysis adjusted for the cofounders sex, age, type of referral and shift was performed to evaluate the association of hCAD with door-to-ECG time.

**Results:**

1101 patients were included in this analysis. 362 patients (33%) had hCAD. Patients with hCAD had shorter door-to-ECG time (20 min. [Inter Quartile Range [IQR] 13–30] vs. 22 min. [IQR 14–37]; *p* < 0.001) when compared to patients with no hCAD. In a multivariable regression analysis hCAD was significantly associated with a shorter door-to-ECG time (− 3 min [*p* = 0.007; 95% confidence Interval [CI] − 5.16 to − 0.84 min]).

**Conclusion:**

In this single center registry, hCAD was associated with shorter door-to-ECG time. In patients presenting in ED for cardiac evaluation, timely ECG diagnostic should be facilitated irrespective of hCAD.

## Background

Chest pain is one of the most common causes for referral to emergency departments (ED) worldwide and is challenging through its heterogeneous causes [1, 2]. Simultaneously, limited access to primary care and patient perceived urgency result in a trend towards increased annual ED attendance [3]. This amounts to an increasing number of patients in need of rapid evaluation to determine whether any life-threatening disease for example one of the “big five” of acute chest pain (aortic dissection, pericarditis with tamponade, esophageal perforation, pulmonary embolus and tension pneumothorax) may be present [4].

In patients with acute myocardial infarction (AMI; “acute myocardial infarction”) a prompt recognition is vital, since the beneficial effects of therapy are greatest when performed soon after symptom presentation [5–7]. Recent clinical evidence emphasize that especially high-risk patients profit from rapid diagnostic and therapy [8, 9]. Therefore, beside clinical history and cardiac markers, early acquisition of a 12-lead electrocardiogram (ECG) in the ED gains importance especially when it comes to the decision for reperfusion therapy. Consequently, a 10 min target for door-to-ECG time is recommended in the majority of national guidelines [7, 8]. Nonetheless, several studies have shown that only one-third of patients with Acute Coronary Syndrome (ACS) receive ECG acquisition attained the target of 10 min after admission. Although societies have made suggestions for performing ECG in the ED, only a minority of the literature addresses how to adhere to the 10 min goal. In the study at hand, we strive to single out clinical factors associated with door-to-ECG time.

Patients with diagnosed coronary artery disease (CAD) have an about 5–10% risk for recurrent cardiovascular events each year and recent guidelines label them to be high-risk patients [10, 11].

Medical history of CAD is part of several risk scores for risk stratification of patients with chest pain and is among the first clinical data the physician would be confronted with after admission. Therefore we suspected hCAD to influence clinical management [12–15].

This single-center, retrospective cohort study aimed to evaluate secular trends in ED workflow, comparing patients with hCAD and patients with no hCAD admitted for chest pain evaluation for door-to-ECG time and time from beginning of symptoms to admission and time to coronary angiography (CAG).

## Methods

### Cohort

In this single center, retrospective cohort study we screened patients admitted to the ED for cardiac evaluation between April and December 2013 for door-to-ECG-time and in a subgroup analysis door-to-CAG-time. Patients were grouped for hCAD and no hCAD accordingly. We also compared both groups in a subgroup analysis of patients who received CAG. The protocol of this study conforms to the ethical guidelines of the 1975 Declaration of Helsinki and was henceforth approved by the institutional ethical committee of University of Freiburg (permit numbers EK99/17).

### Outcomes

Primary outcome was the door-to-ECG-time in all included patients. Secondary outcome was the time from initial symptoms to admission and in patients receiving CAG the door-to-CAG-time.

### Screening

Full-text keyword-search of the anonymized ED Database segments: key symptoms at admission, anamnesis and diagnosis; was used to single out patients admitted to ED for cardiac evaluation. Keywords included: chest pain; dyspnoea; angina pectoris (AP); retrosternal chest pain; shortness of breath; ST-elevation myocardial infarction (STEMI); non-ST-elevation myocardial infarction (NSTEMI); acute myocardial infarction (AMI); Acute coronary syndrome; myocardial infarction and heart failure.

### Patient characteristics and time points

All patient clinical characteristics as well as laboratory data were obtained retrospectively from the hospital's electronic database. Baseline Characteristics include age, sex and cardiovascular risk factors such as pre-existing diagnoses of diabetes mellitus, hypertension, smoking and family with history of cardiovascular disease.

The time points: “start of symptoms”, admission to ED”, “ECG at ED” and “Coronary angiography” were assessed using electronic records or documentation in the hospital database.

### Admission process

Patients admitted to ED are referred to a triage area upon arrival for identifying high and low-urgency patients. Patients admitted via emergency doctor skip this process and are immediately transferred to the ward. Assessment by the hospital triage nurse includes sex, age, chief complaint and epitome of the patient history.

If the emergency doctor suspects a transmural myocardial infarction, the patient is referred directly to the catheter lab for acute coronary intervention bypassing the ED.

### Statistical consideration

Continuous patient data were compared using a T-test if found to follow a Gaussian distribution otherwise data underwent a Mann–Whitney U-test. Categorical differences between patient groups were compared using Fishers exact test. Continuous variables are presented as median ± lower and upper quartiles if found to follow a non-Gaussian distribution and as mean ± standard deviation if found to follow a Gaussian distribution according to the D'Agostino-Pearson omnibus normality test. Categorical patient characteristics are presented as percentages. A multivariable median regression model was established to assess influence of a hCAD on time to ECG. As potential confounders, we took into consideration a predefined number of factors that would be obvious to the caregivers upon patients’ presentation: sex, age and type of referral. We also checked for differences in door-to-ECG time during the different shifts.

As there was no prespecified plan to adjust for multiple comparisons, 95% confidence intervals were not adjusted for multiple comparisons and inferences drawn from them may not be reproducible. Descriptive analyses were performed using Graph Pad Prism Version 6.0 (Prism 6 for Mac OS X; GraphPad Software, Inc., La Jolla, CA) and multivariable median regressions were conducted using Stata version 16.1 (StataCorp, College Station, Texas).

## Results

### Baseline characteristics

1101 patients met the inclusion criteria. Of those, 362 (33%) had hCAD and 739 (67%) had no hCAD. Of those, 351 patients received CAG (172 [49%] patients with hCAD and 179 [51%] patients with no hCAD).

Of all included patients, the ones with hCAD were older than patients with no hCAD (74 years [IQR 65–82] vs. 60 years [IQR 46–74]; *p* < 0.001). 31% in the known-CAD group and 46% in the no-known-CAD group were female (*p* < 0.001).

Compared with patients with no-hCAD, patients with hCAD had higher risk-scores (Global Registry of Acute Coronary Events [GRACE] score: 125 points [IQR 104–147] vs. 93 points [IQR 62–125]; *p* < 0.001 and Thrombolysis In Myocardial Infarction,[TIMI] Score 2 points [IQR 2–3] vs. 1 point [IQR 0–2]; *p* < 0.001), more cardiovascular risk factors (Diabetes mellitus 28% vs. 14%; *p* < 0.001, hypercholesterinaemie 51% vs. 13%; *p* < 0.001, arterial hypertension 80% vs. 44%; *p* < 0.001 and family hCAD 18% vs. 13%; *p* < 0.001) and more co-morbidities (chronic kidney disease 26.8% vs. 4.5%; *p* < 0.001, history of stroke 11.4% vs. 3.7%; *p* < 0.001, peripheral arterial disease 12.9v% vs. 2.3%; *p* < 0.001, heart failure 12.1% vs. 1.5%; *p* < 0.001).

Patients with hCAD were more likely to be referred to the hospital by emergency medical services (238 patients [66%] vs. 362 patients [49%]; *p* < 0.0001) while patients with no hCAD more frequently were self-referrals (56 patients [15%] vs. 223 patients [30]; *p* < 0.001).

### Analysis of time intervals

#### Pre-admission

There was statistical significant difference in the time from initial symptoms to ED admission between patients with hCAD and patients with no-hCAD (0.8 h [IQR 0.6–11] vs. 12 h [5–18]; *p* < 0.001). Patients with hCAD were more likely to receive a pre-clinical ECG (31.8% [N115] vs. 20.7% [N153]; *p* < 0.001) (Table 1).

**Table 1:** (A) Baseline characteristics of included patients, (B) door-to-ECG time in included patients in general, in patients referred via emergency doctors, ambulance services and self-referral respectively

N	No hCAD		HCAD		*p*
	739		362		
*(A)*					
Age in years	60	(46–74)	74	(65–82)	< 0.001^a^
Grace score	93	(62–125)	125	(104–147)	< 0.001^a^
Creatinin in mg/dl	0.9	(0.8–1.1)	1.1	(0.9–1.4)	< 0.001^a^
Female in % (N)	45.7	(338)	31.2	(113)	< 0.001^b^
Pre hospital ECG in % (N)	20.7	(153)	31.8	(115)	< 0.001^b^
Diabetes mellitus in % (N)	13.7	(101)	27.9	(101)	< .0001^b^
Arterial hypertension % (N)	44.1	(326)	79.6	(288)	< 0.001^b^
Chronic kidney failure % (N)	5.1	(38)	25.7	(93)	< 0.0001^b^
Positive family history for CV events in % (N)	13	96	18.2	(66)	0.02^b^
Hyperlipidaemia in % (N)	12.7	94	51.4	(186)	< 0.001^b^
History of smoking in % (N)	23.7	175	20.2	(73)	0.2^b^
TIMI score	2	(2–3)	1	(0–2)	< 0.0001^a^
Peripheral artery disease in % (N)	2	(16)	14	(49)	< 0.0001^b^
Emergency admission^c^ in % (N)	49	(362)	66	(238)	< 0.001^b^
Self referral in % (N)	30	(223)	15	(56)	< 0.001^b^
Time point of admission					
Morning shift in % (N)	42	(308)	48	(175)	0.04^b^
Night shift in % (N)	20	(150)	18	(65)	0.4^b^
Late shift (in % (N)	38	(281)	34	(122)	0.2^b^
*(B)*					
Time-to-EKG in minutes	22	(14–37)	20	(13–30)	< 0.001^a^
Via emergency doctor time-to-EKG in minutes	2	(0–15)	16	(12–23)	< 0.001^a^
Via ambulance service time-to-EKG in minutes	20	(12–35)	19	(14–25)	< 0.001^a^
Self-referral time-to-EKG in minutes	32	(20–51)	30	(16–49)	0.5^a^
Total time in CPU in hours	6.4	(5–11)	7.5	(5–10)	< .0001^a^
First symptoms to admission in hours	12	(5–18)	0.8	(0.6–11)	< 0.001^a^

N = Number of patients; *p* values refer to the comparison between the hCAD negative and the hCAD positive patients

^a^Presented as median ± interquartile range

^b^Based on chi-square test/Fisher’s exact test as appropriate for categorical variables

^c^Referred via Emergency Doctor, Rescue services, airborn rescue services/air rescue

#### Following admission to ED

**Fig. 1:**
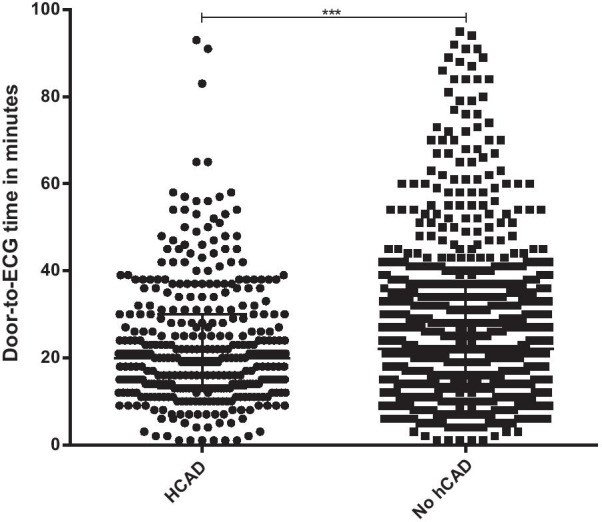
Time to ECG in patients with hCAD and no hCAD respectively. Data are presented as Scatter blocks with median and interquartile range. ***p < 0.001; *CAD* coronary artery disease

The total time in the ED was significantly higher for patients with known hCAD when compared with patients with no known hCAD (7.5 h [IQR 5–10] vs. 6.4 h [IQR 5–11]; *p* < 0.001). Patients with known hCAD had significant shorter door-to-ECG time after admission when compared to patients with no known hCAD (20 min [IQR 13–30] vs. 22 min [IQR 14–37]; *p* < 0.001) (Fig. 1).

There was a statistically significant difference in door-to-ECG time during different shifts: Night- and early-shift (− 4.85 min. [*p* < 0.001; 95 CI − 7.39 to − 2.32]). (Table 3).

Patients with known hCAD were more likely to be admitted during the early shift (48% [N175] vs. 42% [N308]; *p* = 0.04) (Table 2).

**Table 2:** (A) Baseline characteristics of patients who received coronary angiography, (B) outcome of patients who received coronary angiography

N	No hCAD		HCAD		*p*
	179		172		
*(A)*					
Age in years	67	(57–75)	73	(63–80)	< 0.001^a^
Troponin T µg/L at admission	0.02	(0.01–0.15)	0.02	(0.01–0.05)	0.3^a^
GRACE score	108	(88–134)	125	(105–150)	< 0.001^a^
Male in % (N)	52	(93)	70	(121)	< 0.001^b^
TIMI score	1.9	(± 1.1)	2.4	(± 0.93)	< 0.001^c^
*(B)*					
Admission to coronary angiography in hours	24.5	(5–54)	33.3	(9–68)	0.01^**a**^
Results of angiogram					
Percutaneous transluminal coronary angioplasty in % (N)	42	(76)	45	(79)	0.4^b^
Percutaneous coronary intervention in % (N)	42	(76)	44	(76)	0.8^b^
Number of stents	1	(1–2)	1	(1–2)	0.7^a^
Number of involved vessels	2	(1–3)	3	(2–3)	0.004^a^
Stable disease/exclusion of relevant CAD in % (N)	56	(101)	53	(91)	0.5^b^
NSTEMI in % (N)	25.1	(45)	36	(62)	0.02^b^
NSTEMI admission to coronary angiography in hours	20.7	(6–33)	29.5	(10–48)	0.01^a^
STEMI in % (N)	8.4	(15)	7.5	(6)	0.8^b^
NSTEMI patients CK-max. (U/I) within 48 h after admission	357	(162–791)	245	(110 - 385)	0.03^b^

N = Number of patients; *p* values refer to the comparison between the hCAD negative and the hCAD positive patients

*CAD* coronary artery disease, *NSTEMI* non-ST segment elevation myocardial infarction, *STEMI* ST-segment elevation myocardial infarction, *CK* creatinkinase

^a^Presented as median ± interquartile range

^b^Based on chi-square test/Fisher’s exact test as appropriate for categorical variables

^c^Presented as mean ± standard deviation

#### Multivariable regression analysis

Multivariable regression analysis of all patient data with the dependent variable being door-to-ECG time showed a relevant association of hCAD with door-to-ECG time with a coefficient of − 3 min (*p* = 0.007; 95% CI − 5.16 to − 0.84 min). This association prevailed when only patients admitted via health care professionals (e.g. emergency doctor) were considered with − 3.9 min (n = 598; *p* = 0.006; 95% CI − 6.7 to − 1.1 min).

Data analysis also showed that patient age correlated with delay in door-to-ECG time (0.007 min [*p* = 0.012; 95% CI 0.02–0.13]). Admission to ED via emergency services was associated with a shorter time to ECG (− 3.53 min [95% CI − 5.69 to − 1.38 min; *p* = 0.001]) (Table 3).

**Table 3:** Multivariable median regression analysis (A: all patients. N = 1.101; B: Emergency patients^a^ only, N = 598). Dependent variable: Door-to-ECG time

	Coefficient	*p* value	95% CI	
*(A)*				
HCAD	− 3.00	0.007	− 5.16	− 0.84
Emergency admission^a^	− 3.53	0.001	− 5.69	− 1.38
Early shift	Reference			
Night shift	− 4.85	< 0.001	− 7.39	− 2.32
Late shift	− 1.76	0.159	− 4.20	0.69
Female	0.48	0.659	− 1.65	2.61
Age	0.07	0.012	0.02	0.13
*(B)*				
HCAD	− 3.93	0.006	− 6.74	− 1.12
Early shift	Reference			
Night shift	− 2.67	0.154	− 6.36	1.01
Late shift	− 1.95	0.249	− 5.28	1.37
Female	0.77	0.608	− 2.17	3.70
Age	0.05	0.229	− -0.03	0.12

^a^Referred via Emergency Doctor, Rescue services, airborn rescue services/air rescue

If only patients admitted via emergency services were considered (N = 598), hCAD was the only factor associated with a shorter door-to-ECG time (− 3.93 min [*p* = 0.006; 95% CI − 6.74 to − 1.12]) (Table 3B).

#### Outcome analysis

351 patients were referred to CAG after ED admission, 179 (51%) with hCAD and 172 (49%) with no hCAD.Patients with hCAD referred to CAG were more likely to be male when compared to patients with no hCAD (70% [121 N] vs. 52% [93]; *p* < 0.001). There was no statistically significant difference in number of percutaneous transluminal coronary angioplasty (42% [76 N] vs.45% [79 N]; *p* = 0.4) or percutaneous coronary intervention (42% (76 N) vs. 44% [75 N] *p* = 0.8) performed in patients with no hCAD and hCAD accordingly. Patients with hCAD were statistically significantly more often discharged with the final diagnose being NSTEMI (45% [62 N] vs. 25% [36 N]). NSTEMI patients with no hCAD had a statistically significant higher levels of creatinkinase within 48 h after admission (357 U/I [162–791] vs. 245 U/I [110–385]; *p* = 0.03). There was no statistically significant difference in patients with no hCAD being discharged with the diagnose “Stable Disease” or “Exclusion of relevant CAD” (53% (91 N) vs. 56% [101 N]; *p* = 0.5). Patients with hCAD had a statistically significant longer door-to-CAG time (33.3 h [IQR 9–68] vs. 24.5 h [IQR 5–54]; *p* = 0.01) when compared to patients with no hCAD. This statistically significant difference prevailed when looking at patients with final diagnose: NSTEMI (29.5 h [IQR 10–48] vs. 20.7 h [IQR 6–33]; *p* = 0.01) (Table 2).

## Discussion

In this single center, retrospective, observational registry, we evaluate the influence of a known hCAD on door-to-ECG time in patients referred to the ED for cardiac evaluation and in a subgroup analyses of patients transferred to CAG, on door-to-CAG time when compared to CAD-naïve patients.

We show that hCAD is associated with a decrease in door-to-ECG time especially in patients admitted via emergency services. This increased awareness for recurrent cardiovascular events in medical personnel might be due to the frequency healthcare professionals are confronted with cardiovascular disease. This presumption is validated by several studies showing that patients with a history of CV events are more likely to experience a recurrent CV event especially since they often have an elevated cardiovascular risk profile. It is vital to understand what influences door-to-ECG time after emergency admission to ED as studies show, that door-to-ECG time is one of the main controllable factors influencing door-to-balloon time [16, 17].

Especially since the new guidelines push for an increased awareness of high-risk NSTEMI patients who are eligible for fast-track coronary angiography, a rapid ECG after ED admission gained a central role in the decision for early reperfusion therapy and a 10 min rule for door-to-ECG time is recommended in guidelines [7]. Our results show a significantly reduced door-to-ECG time when compared to other studies which may be due to the implementation of a chest pain unit (CPU) into ED management in early 2013 [18]. However, still only a fraction received ECG within the suggested time frame especially in self-referral patients [19]. This calls for an increased effort for timely ECG in the ED irrespective of hCAD but may be associated with the fact that a relevant part of included patients received pre-clinical ECGs via the emergency doctor and that in self-referral patients we included the time of triage.

Also associated with door-to-ECG time was patient age with older patients experiencing a delay in door-to-ECG time by the year. Several studies have shown, that older patients are at risk to receiving an assignment of an inappropriately low triage level possibly due to different reference values of vital signs, atypical disease presentations, or the presence of cognitive impairment [20]. Nevertheless rapid diagnostic in ED should be facilitated irrespective of age.

Our data also shows, that in patients with hCAD the referral to subsequent coronary angiography is prolonged despite an increased GRACE score suggesting an early invasive strategy.

This delay in invasive diagnostic might be owed to the fact that patients with hCAD presented with a more complex array of not only cardiovascular risk factors but relevant comorbidities which makes it more difficult for the clinician to get an overview on the patient history and put his current symptoms into perspective [21]. Nonetheless, as those patients presented with a higher TIMI- and GRACE score, they were likely to profit from a more rapid approach. This shows that risk score assessment might be underutilized in ED despite being a useful tool to single out high-risk patients eligible for fast-line diagnostic [12, 13].

Also, in accordance with other studies, our results show that patients with renal impairment are less likely to receive early CAG. This is most likely of mixed genesis including an uncertainty as to the interpretation of troponin measurements in patients with chronic kidney disease (CKD), atypical presentation of symptoms and concerns regarding acute kidney failure after contrast medium induced acute kidney injury [22–24]. Nevertheless, international guidelines also support an early invasive management strategy in CKD patients with suspected AMI due to a two–fivefold greater risk of death after AMI and ED personnel should be briefed accordingly [25].

As a result of the aforementioned delayed referral to CAG, it is incidental that patients with hCAD spent longer time at the ED. Reducing the length of stay through accelerating door-to-ECG time in all patients could be a powerful tool to cost saving in ED [26].

Our data show, that patients with hCAD had increased likelihood for acute myocardial infarction when compared with patients with no hCAD. This is supported by findings in other studies [27]. It may be partly explained by the fact that patients with hCAD had more cardiovascular risk factors and understandable since patients with hCAD had higher probability for recurrent ischemic events determent by TIMI- and GRACE score [13]. Nevertheless, hCAD is easily retrievable information for patient triage at ED.

We suspected and there are studies elaborating, that patients with hCAD due to the fact that they are able to identify symptoms quicker and arrive at ED faster when compared to CAD naïve patients [28]. Consequently, our data show a statistically significant difference between patients with hCAD and patients with no hCAD in respect to time from beginning of symptoms to admission to ED. Arguably, this might be due to the fact that patients with a hCAD are eligible to recall classical symptoms such as angina pectoris faster and act accordingly.

A similar picture emerges when we examined the modus of referral to the hospital in the different groups with patients with hCAD being far more likely to call and be referred by emergency medical services speaking for an increased awareness in those patients. Consequently those patients were more likely to receive a preclinical ECG, which might improve their outcome as some studies suggest [16].

Finally, our results show, that door-to-ECG time also depends on the shift during which patients are admitted to the ED. Especially at night with a smaller number of patients admitted door-to-ECG is significantly reduced. Nevertheless, in patients referred via emergency services, hCAD proofed to be the only factor statistically significant associated with shorter door-to-ECG time.

## Limitations

Based on the presented results we plan to initiate an interventional study to improve door-to-ECG time in self-referral patients. This is a retrospective observation and we tried to depict all day routine in ED. Consequently we didn’t differentiate between acute coronary syndrome and chest pain. The majority of patients with ST-myocardial infarction are not admitted via ED but directly in the cardiac catheterization laboratory therefore we could not include them. The time frame concerning begin of symptoms was taken from patients memory and is subject to individual deviations. We did include all patients admitted to ED and tried to select via full-text search, however there is still the possibility that patients were admitted for e.g. neurological or orthopaedic diagnostic and developed symptoms in the ED. All patients had hCAD but differ in presentation. That means we also included patients who did undergo elective CAG for example through initiation of a cardiologist. Therefore, such patients wouldn't know the symptoms of ACS. We did not include regression analysis for influence of hCAD on time-to-CAG since there would have been too many confounders.

## Conclusion

Our observational data from a single centre registry show, that hCAD prior admission to ED is associated with a shorter door-to-ECG time. Although patients with hCAD were more often high-risk patients with a higher GRACE score, more co-morbidities and a higher cardiovascular risk profile and would therefore benefit from an early invasive strategy, they are referred to CAG later. Whether this influences patient outcome needs to be evaluated in further clinical trials. In patients referred to the ED for cardiac evaluation, timely ECG diagnostic should be facilitated irrespective of hCAD.

## Data Availability

The datasets generated for this study are available on reasonable request to the corresponding author.

## Abbreviations

ED: Emergency department
ECG: Electrocardiogram
CAD: Coronary artery disease
hCAD: History of coronary artery disease
CAG: Coronary angiography
AMI: Acute myocardial infarction
PAD: Peripheral artery disease
FMC: First medical contact
CV: Cardiovascular
